# Cecropin A Modulates Tight Junction-Related Protein Expression and Enhances the Barrier Function of Porcine Intestinal Epithelial Cells by Suppressing the MEK/ERK Pathway

**DOI:** 10.3390/ijms19071941

**Published:** 2018-07-02

**Authors:** Zhenya Zhai, Xiaojun Ni, Chenglong Jin, Wenkai Ren, Jie Li, Jinping Deng, Baichuan Deng, Yulong Yin

**Affiliations:** 1 Guangdong Provincial Key Laboratory of Animal Nutrition Control, Subtropical Institute of Animal Nutrition and Feed, College of Animal Science, South China Agricultural University, Guangzhou 510642, Guangdong, China; zhai@stu.scau.edu.cn (Z.Z.); nnnxjun@stu.scau.edu.cn (X.N.); jin@stu.scau.edu.cn (C.J.); renwenkai19@126.com (W.R.); lijiedk@stu.scau.edu.cn (J.L.); 2 National Engineering Laboratory for Pollution Control and Waste Utilization in Livestock and Poultry Production, Institute of Subtropical Agriculture, The Chinese Academy of Sciences, Changsha 410125, Hunan, China

**Keywords:** antimicrobial peptide, cecropin A, tight junction protein, MEK/ERK signaling, porcine intestinal epithelial cell

## Abstract

Inflammatory bowel disease (IBD) in humans and animals is associated with bacterial infection and intestinal barrier dysfunction. Cecropin A, an antimicrobial peptide, has antibacterial activity against pathogenic bacteria. However, the effect of cecropin A on intestinal barrier function and its related mechanisms is still unclear. Here, we used porcine jejunum epithelial cells (IPEC-J2) as a model to investigate the effect and mechanism of cecropin A on intestinal barrier function. We found that cecropin A reduced *Escherichia coli* (*E. coli*) adherence to IPEC-J2 cells and downregulated mRNA expression of tumor necrosis factor α (*TNF*-*α*), interleukin-6 (*IL*-*6*), and interleukin-8 (*IL*-*8*). Furthermore, cecropin A elevated the transepithelial electrical resistance (TER) value while reducing the paracellular permeability of the IPEC-J2 cell monolayer barrier. Finally, by using Western blotting, immunofluorescence and pathway-specific antagonists, we demonstrated that cecropin A increased ZO-1, claudin-1 and occludin protein expression and regulated membrane distribution and F-actin polymerization by increasing CDX2 expression. We conclude that cecropin A enhances porcine intestinal epithelial cell barrier function by downregulating the mitogen-activated protein kinase (MEK)/extracellular signal-regulated kinase (ERK) pathway. We suggest that cecropin A has the potential to replace antibiotics in the treatment of IBD due to its antibacterial activity on gram-negative bacteria and its enhancement effect on intestinal barrier function.

## 1. Introduction

Anti-infective drugs play important roles in the prevention and treatment of inflammatory bowel disease (IBD) in humans and animals. IBD is a complex gastrointestinal disease, mainly induced by infection with gram-negative bacteria such as *Escherichia coli* (*E. coli*) and *Salmonella* [1]. In recent decades, an increasing prevalence of antibiotic resistance has threatened human health [2]. Finding effective alternatives to antibiotics has become an increasingly urgent task. Among the potential alternatives, antimicrobial peptides (AMPs) are particularly important, due to their broad spectrum antibacterial activity and decreased likelihood of inducing antibiotic resistance. Currently, more than 2800 AMPs have been found in animals, plants or microorganisms [3]. In animals, AMPs play important roles in host defense and are crucial in the immune system [4]. The activities of AMPs vary greatly due to their different sequences and structures. In addition to antimicrobial activity, some AMPs also have wound healing abilities through promoting cell proliferation, reducing inflammation or enhancing intestinal barrier function [5,6].

Cecropins are a group of peptides with an α-helical structure and were initially found in insects. Currently, there are more than 30 records of cecropins in the Antimicrobial Peptide Database (APD), including naturally discovered and artificially synthesized cecropins [3]. Cecropin A is one of the earliest discovered cecropins by Steiner et al. from *Hyalophora cecropia* [7]. Over the past decades, the antibacterial mechanisms of cecropin A have been extensively researched [8,9]. In addition, cecropin A is also a commonly used template for peptide molecular hybrids to enhance the antibacterial activity of AMPs [10]. Although the antibacterial activity of cecropin A has been demonstrated for decades, to our best knowledge, the effect of cecropin A on intestinal barrier function is still unknown. 

IBD is caused by pathogenic bacterial infection and intestinal mucosal barrier disruption. Intestinal mucosal surfaces consist of epithelial cells, such as absorptive cells, endocrine cells and Paneth cells [11]. The epithelial cells form a selectively leaky barrier, which is crucial for nutrient substance exchange and host defense [12,13,14,15]. These functions depend on intact intestinal epithelial cell layers, which are composed of cell–cell attachments at the cell lateral membrane by tight junctions (TJs) and subjacent adherens junctions [13]. The TJs consist of transmembrane proteins such as claudins, occludin and junctional adhesion molecules (JAMs). These proteins are clustered and stabilized by cytoplasmic scaffolding proteins called zonula occludens (ZOs) and cytoskeletons such as F-actin. Different tight junction proteins play various roles in barrier function. Claudins and occludin are located at apical and basal positions of the lateral membrane, respectively [16]. ZOs, such as ZO-1, can interact with cytoskeleton, claudin-1 and occludin [17]. To summarize, the TJ-cytoskeleton structure is essential for the intestinal barrier.

The regulation of TJ expression and membrane distribution is complex. The mitogen-activated protein kinase (MAPK) pathways, which contain three downstream pathways including extracellular signal-regulated kinase (ERK), p38 and c-jun, are responsible for cell proliferation, proliferation and immune reaction in the gastrointestinal tract [18]. ERK, which may be activated by mitogen-activated protein kinase (MEK), is one of the most important pathways for maintaining gastrointestinal tract homeostasis and regulating the intestinal barrier. However, according to previous studies, the effect of MEK/ERK on intestinal barrier function is controversial. Piegholdt et al. [19] showed that biochanin A and prunetin may improve epithelial function through downregulation of ERK, while Wang et al. [20] showed an improvement of the intestinal epithelial barrier through upregulation of the ERK pathway by polyphenol-rich propolis extracts. The regulatory effect of the MEK/ERK signaling pathway on the intestinal barrier is unclear.

In this study, we evaluated the effects of cecropin A on intestinal barrier function in an IPEC-J2 cell monolayer model. We also detected the TJ protein level and membrane distribution by using Western blotting and cell immunofluorescence, respectively. Finally, the changes in the MEK/ERK signaling pathway were detected to reveal the regulatory mechanism of cecropin A on the barrier function.

## 2. Results

### 2.1. The Antibacterial Activity of Different AMPs

Seven AMPs were selected from the APD database, antibacterial activities including minimum inhibitory concentration (MIC) and minimum bactericidal concentration (MBC) were tested by using 11 specific bacterial strains (Table S1) and the information of origin source, peptide length, net charge were also described (Table S2). The results showed that cecropin A possessed the best antibacterial activity (MIC and MBC between 1.5 and 6.25 μg/mL) to gram-negative bacterial strains, such as *E. coli*, *Salmonella* and *Pseudomonas aeruginosa*.

### 2.2. Cytotoxicity to IPEC-J2 Cells

The cytotoxicity of AMPs to the pig intestinal epithelium cell IPEC-J2 was evaluated by using an MTT (3-(4,5-dimethylthiazol-2-yl)-2,5-diphenyltetrazolium bromide) assay (Figure S1A–G). The concentration was 1.5–100 μg/mL. The results showed that IPEC-J2 cell viability was not reduced after treatment with cecropin A (1.5–12.5 μg/mL, Figure S1C) for 8 h compared to that of the control group. Six other AMPs reduced cell viability in a dose-dependent manner.

### 2.3. Cecropin A Inhibits E. coli Adherence and Ameliorates Inflammation

**Figure 1:**
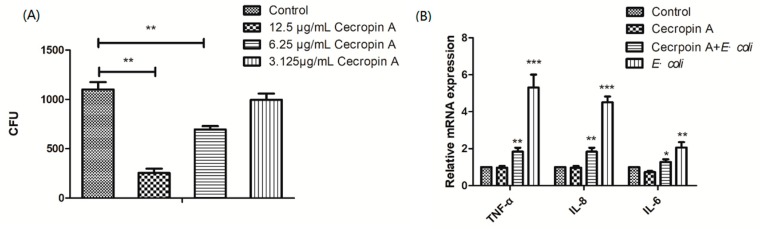
Cecropin A inhibited bacterial adherence and reduced the expression of inflammatory factors. The porcine jejunum epithelial cells (IPEC-J2) cells were pretreated with cecropin A (3.125, 6.25, and 12.5 μg/mL) for 48 h, and the CFU of adherent bacteria were counted ((**A**), *n* = 3). The relative mRNA expression of *TNF*-*α*, *IL*-*8*, and *IL*-*6* were tested by using qPCR ((**B**), *n* = 6). Control: control group; cecropin A: cells were pretreated with cecropin A; cecropin A + *E. coli*: cells were pretreated with cecropin A and then cocultured with *E. coli*; *E. coli*: cells cocultured with *E. coli*. The results were confirmed by three independent experiments per treatment. Representative results of the three independent experiments are shown. Data (mean ± SEM) were analyzed with one-way ANOVA. * *p* < 0.05, ** *p* < 0.01, *** *p* < 0.001.

IPEC-J2 cells pretreated with 3.125, 6.25, and 12.5 μg/mL cecropin A displayed reduced *E. coli* adherence in a dose-dependent manner (Figure 1A). In addition, after coculture with *E. coli*, the TNF-α, IL-6, and IL-8 mRNA expression in IPEC-J2 cells was also downregulated after cecropin A treatment for 48 h, compared to that in the non-treated cells (Figure 1B). The results suggested that the defense capability of IPEC-J2 cells against bacteria was increased.

### 2.4. Cecropin A Increases the TER and Decreases the Paracellular Diffusion of FITC-Dextran through the IPEC-J2 Monolayer

**Figure 2:**
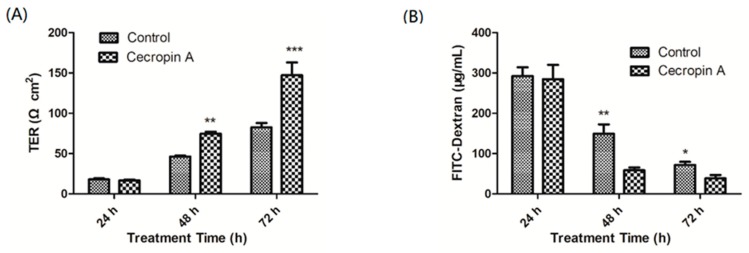
Cecropin A enhances the transepithelial electrical resistance (TER) and reduces the permeability of the IPEC-J2 cell monolayer. The TER ((**A**), *n* = 6) and permeability ((**B**), *n* = 6) of the cell monolayer were tested after treatment with cecropin A for 24 h, 48 h, and 72 h. The results were confirmed by three independent experiments per treatment. Representative results of the three independent experiments are shown. Data (mean ± SEM) were analyzed with the Student’s *t*-test. * *p* < 0.05, ** *p* < 0.01, *** *p* < 0.001.

The integrity of the intestinal barrier may increase the defense capability of the host and reduce the adherence of pathogenic bacteria. We examined whether cecropin A could enhance the intestinal monolayer barrier function. Transepithelial electrical resistance (TER) values were assessed at 24, 48 and 72 h after cecropin A treatment. Our data showed that, compared to those of the control cells, the TER values of cecropin A-treated cells were significantly increased at 48 h and 72 h (Figure 2A; *p* < 0.01). In addition, we measured the permeability of large solutes by using 4 kDa FITC-dextran as a tracer. The data showed that, after cell incubation with FITC-dextran, the concentrations of FITC-dextran in the basal compartment were significantly higher than those in cecropin A-treated cells at 48 h and 72 h (Figure 2B, *p* < 0.05).

### 2.5. Cecropin A Regulates TJ Protein Expression Levels, Membrane Distribution and F-Actin Polymerization

**Figure 3:**
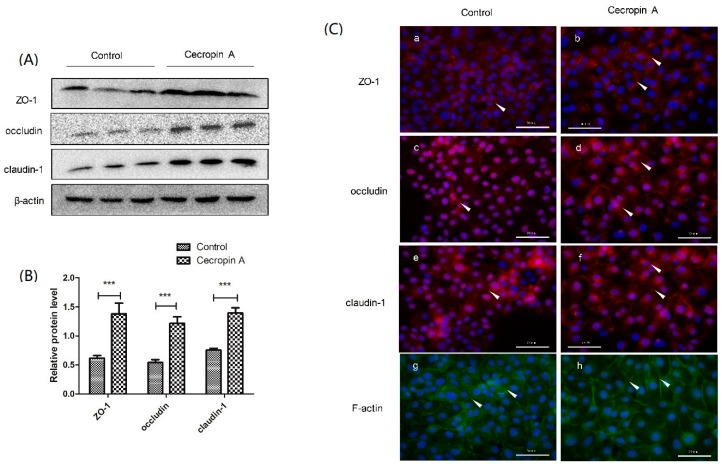
The effect of cecropin A on tight junction (TJ) protein expression, membrane distribution and F-actin polymerization. Western blotting analysis of zonula occludens-1 (ZO-1), occludin and claudin-1 expression were upregulated by cecropin A treatment ((**A**), *n* = 3); quantification of ZO-1, occludin, and claudin-1 protein expression was shown ((**B**), *n* = 3); cell immunofluorescence (**C**) (400×) showed that the cecropin A induced the TJs polymerized at the cell–cell boundary (**a**–**f**) and indicated F-actin polymerization (**g**,**h**) in cells ((**c**), *n* = 3). The results were confirmed by three independent experiments per treatment. Representative results of the three independent experiments are shown. Data (mean ± SEM) were analyzed with the Student’s *t*-test. Cell nuclei were stained by 4′,6-Diamidino-2-phenylindole dihydrochloride (DAPI) and are shown in blue. Claudin-1, ZO-1 and occludin are shown in red and pointed out by white arrow heads. F-actin is shown in green. Scale bar is 50 μm. *** *p* < 0.001.

To elucidate how cecropin A increases the TER value and decreases paracellular permeability, we measured the protein levels of ZO-1, claudin-1 and occludin. The results showed that the protein levels of TJs were significantly upregulated (Figure 3A,B). We also detected TJ membrane distribution by using cell immunofluorescence. The results showed that ZO-1, claudin-1 and occludin were much more polymerized at the cell–cell boundary in the cecropin A group than in the control group. (Figure 3C). The integrity of the intestinal monolayer barrier is coordinated by the connection between TJs and the cytoskeleton. Representative F-actin staining indicated that the cecropin A group had more extensive F-actin than the control group (Figure 3C). In addition, F-actin was much more polymerized at the cell–cell boundary in the cecropin A group than in the control group. In accordance with the TJ distribution, the results suggested that the better organized F-actin-TJ structure may help increase the monolayer barrier function and the defense capability against the adherence of *E. coli*.

### 2.6. Cecropin A Regulates the Intestinal Barrier by Downregulating the MEK/ERK Pathways

**Figure 4:**
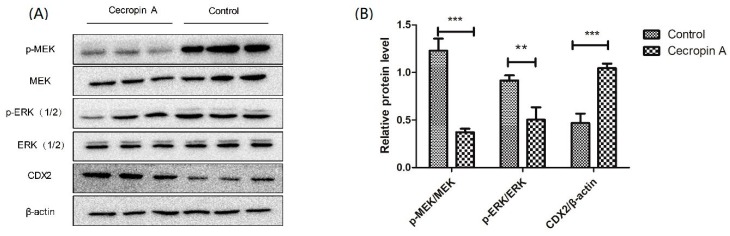
Cecropin A downregulates mitogen-activated protein kinase (MEK)/extracellular signal-regulated kinase (ERK) phosphorylation and upregulates CDX2 expression, *n* = 3. Western blotting analysis of p-MEK, MEK, p-ERK(1/2), ERK (1/2) and CDX2 showed that cecropin A downregulated the MEK/ERK pathway and increased CDX2 protein level (**A**,**B**). The results were confirmed by three independent experiments per treatment. Representative results of the three independent experiments are shown. Data (mean ± SEM) were analyzed with the Student’s *t*-test. Control: control group; Cecropin A: cells treated with cecropin A group. ** *p* < 0.01, *** *p* < 0.001.

To elucidate how cecropin A regulates the TER and TJs, the MEK/ERK signaling pathway was detected using Western blotting. The data showed that phosphorylation of MEK and ERK in control group cells was significantly higher than that in the cecropin A group cells (*p* < 0.01, Figure 4). Caudal type homeobox 2 (CDX2), a transcriptional factor that regulates the differentiation of intestinal cells, was also detected (*p* < 0.001). The results showed that the CDX2 protein level was upregulated in the cecropin A group compared with that in the control group. In addition, the results suggested that cecropin A may regulate TJ expression through regulating the phosphorylation of MEK and ERK and the expression of CDX2.

### 2.7. Inhibition of the MEK/ERK Pathway Increases TER, TJ Expression, Membrane Distribution and F-Actin Polymerization

**Figure 5:**
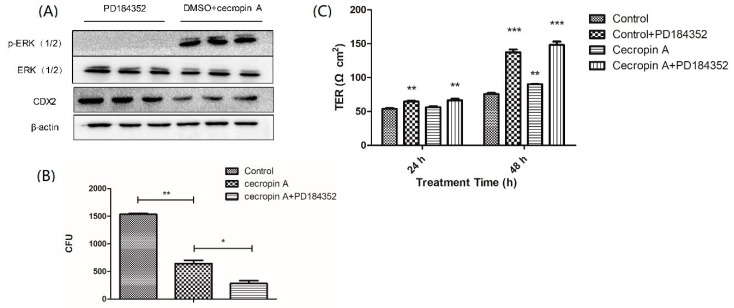
The effect of inhibiting ERK phosphorylation on TER and *E. coli* adherence. TER was upregulated ((**B**), *n* = 3) through inhibition of ERK phosphorylation ((**A**), *n* = 3). IPEC-J2 cells were treated with the ERK-specific inhibitor PD184352, cecropin A and cecropin A + PD184352 for 24 and 48 h, and the CFUs of adherent *E. coli* were decreased after the PD184352 treatment for 48 h (*n* = 3). Data (mean ± SEM) were analyzed with the Student’s *t*-test (**A**) and one-way ANOVA (**B**,**C**). The results were confirmed by three independent experiments per treatment. Representative results of the three independent experiments are shown. * *p* < 0.05, ** *p* < 0.01, *** *p* < 0.001.

**Figure 6:**
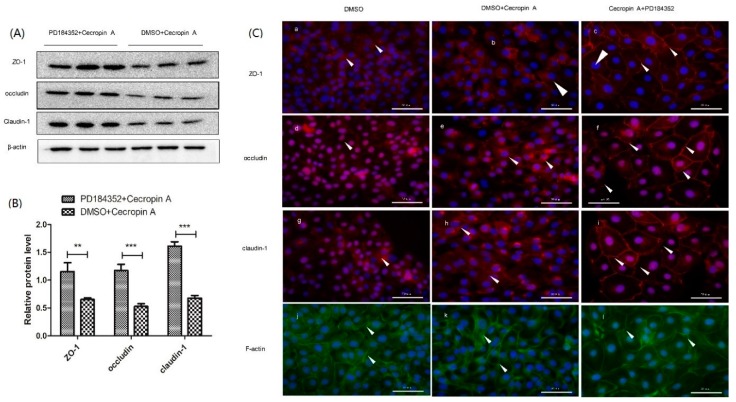
The inhibitory effects of MEK/ERK on TJ protein expression, membrane distribution and F-actin polymerization. Western blotting analysis of ZO-1, occludin and claudin-1 expression ((**A**,**B**), *n* = 3); cell immunofluorescence ((C), *n* = 3, 400×) showed the membrane distribution (**a**–**i**) and F-actin polymerization (**j**–**l**). The results were confirmed by three independent experiments per treatment. Representative results of the three independent experiments are shown. Data (mean ± SEM) were analyzed with the Student’s *t*-test. Cell nuclei were stained by DAPI and are shown in blue. Claudin-1, ZO-1 and occludin are shown in red and pointed out by white arrow heads. F-actin is shown in green. Scale bar is 50 μm. ** *p* < 0.01, *** *p* < 0.001.

To confirm the regulatory effect of the MEK/ERK pathway on the intestinal barrier and TJs, a specific inhibitor (PD184352) of ERK 1/2 was used (Figure 5A). After treatment with the inhibitor for 48 h, the adherence of *E. coli* was reduced compared to that in the cecropin A group (Figure 5B). The TER values of the cecropin A-treated group were significantly higher than those of the control group, while the TER values of the PD184352 + cecropin A group were higher than those of the cecropin A group (Figure 5C, *p* < 0.01). In addition, protein levels of CDX2, ZO-1, claudin-1, and occludin in the PD184352 + cecropin A group were significantly higher than those in the cecropin A group (*p* < 0.001, Figure 6A,B). Moreover, the TJs and F-actin in the PD184352 + cecropin A group were distributed at the cell-cell adjacent position (Figure 6C). The results showed that the inhibition of MEK/ERK may increase intestinal barrier function by increasing the TJ expression level and membrane distribution.

## 3. Discussion

In recent decades, thousands of AMPs have been found in organisms. The antibacterial activities of AMPs differ greatly from each other because of their different sequences and structures [21,22]. Although several antibacterial theories for AMPs have been put forward, their potential mechanisms are still unclear. According to previous studies, most of the AMPs have hemolytic activity or cytotoxicity when used at high concentrations [23]. In this study, we have shown that cecropin A had better antibacterial activity and lower cytotoxicity compared to those of other AMPs (PG-1, LL-37, etc.) selected from the APD3 database. Interestingly, we also found that the antibacterial activity of cecropin A is much higher against *E. coli*, *Salmonella typhimurium* and *Pseudomonas aeruginosa* than *Staphylococcus aureus*, which may suggest that cecropin A was more effective against gram-negative bacteria.

In addition to the antibacterial activities, we also showed that cecropin A may enhance the barrier function of the IPEC-J2 monolayer and increase the defense capability against pathogenic bacteria. In this study, we showed that after treatment with cecropin A for 48 h, adherent *E. coli* were significantly reduced and the mRNA levels of TNF-α, IL-6 and IL-8 were downregulated, which indicates that inflammation may be alleviated. The integrity of intestinal barrier function and morphology may affect the colonization of harmful bacteria and play an important role in protecting hosts from microorganism infection and inflammatory diseases induced by pathogenesis [24,25]. To evaluate the intestinal barrier function, TER and cell monolayer permeability experiments were performed. The data showed that cecropin A could significantly increase TER and reduce permeability. The TJs (ZOs, occludin, claudins, etc.) are the most important parts of the epithelial barrier and are essential for cell–cell adhesion [13]. Our results showed that protein expression levels of ZO-1, occludin and claudin-1 were increased. In addition, immunofluorescence showed that the TJs we detected were distributed on the cell membranes after cecropin A treatment. ZOs (mainly ZO-1) are connected to the cytoskeleton (F-actin). On the other hand, ZO-1 also connected to the intracellular loops of claudins and occludin. Claudins and occludin are responsible for adjacent cell connections. The cytoskeleton-TJ structure may prevent pathogenic bacterial adherence or invasion into cells. The function of TJs depends on the protein expression level and membrane distribution [26]. In this study, we evaluated the expression and location of claudin-1, ZO-1, and occludin by using cell immunofluorescence. The results showed that the membrane distribution of claudin-1, ZO-1 and occludin was significantly increased by cecropin A stimulation, which suggests that cecropin A may regulate the barrier function through regulation of TJ expression and membrane expression.

To elucidate the mechanism of cecropin A regulating the intestinal monolayer barrier, the MEK/ERK signaling pathway was detected. The results showed that cecropin A may enhance the barrier function, regulate TJ expression and membrane distribution by downregulating the MEK/ERK pathway. The MEK/ERK pathway is conserved among eukaryotes, and one of the most important roles of MEK/ERK is to regulate cell proliferation and inhibit differentiation in epithelial cells and tumor cells [18,21]. In the MEK/ERK signaling pathway, ERK 1/2 may be activated by MEK, and then ERK 1/2 may regulate downstream transcriptional factors and widely regulate cell physiological processes, such as proliferation, differentiation, migration and apoptosis [18]. In addition, previous studies have also showed that MEK/ERK inhibition may induce upregulation of TJs in undifferentiated cells, such as embryonic stem cells, intestinal epithelial cells (IEC-6, caco-2) or tumor cells [27,28]. Similarly, ERK activation may induce blood–brain barrier injury [29]. The related physiological processes are regulated by the transcriptional factors downstream of ERK. CDX2, a caudal-related homeobox gene, is an essential regulator of gene transcription and tumor suppression in gastrointestinal tract development and homeostasis [30,31]. Previous studies have shown that CDX2 may play important roles in cell differentiation and proliferation and that it is regulated by the MEK/ERK signaling pathway [27]. In addition, CDX2 also plays an important role in TJ regulation in the intestinal epithelium. Previously, studies showed that in rat intestinal epithelium-derived line, IEC-6, caco-2 and colorectal carcinoma cells, downregulation of CDX2 by the MEK/ERK signaling pathway may decrease the protein expression levels of claudin-1, occludin and ZO-1 [27,32,33]. Consistent with this, our results showed that cecropin A may downregulate MEK and ERK phosphorylation, upregulate CDX2 expression, and upregulate protein levels of ZO-1, claudin-1 and occludin. Interestingly, previously studies showed that LL-37 and human beta defensin-3 (hBD-3) may activate phosphatidylinositide 3 kinases (PI3K)-Protein kinase B (Akt), PKC (protein kinase C) and Glycogen synthase kinase 3β (GSK-3β) and upregulate TJ expression and membrane distribution in human skin cells [26,34], which may be involved in the cell adherence and junction remodeling pathway, suggesting that more than one pathway exists to regulate TJ expression. Overall, in this study, we found that cecropin A enhanced the barrier function in the IPEC-J2 cell monolayer model by upregulating the TJ protein level (ZO-1, claudin-1 and occludin) and membrane polymerization, which was negatively regulated by the MEK/ERK signaling pathway. Our results suggested that cecropin A has the potential to replace antibiotics in the treatment of IBD due to its antibacterial activity on gram-negative bacteria and enhancement effect on intestinal barrier function.

## 4. Materials and Methods

### 4.1. Bacterial Strains

The *Escherichia coli* ATCC 35401, ATCC 35150, ATCC 25922, and SSI 82000, *Pseudomonas aeruginosa* ATCC 9027 and ATCC 27853, *Salmonella* ATCC13312 and ATCC9120, *Salmonella typhimurium* ATCC 14028, and *Staphylococcus aureus* ATCC 29213 strains were purchased from Guangdong Culture Collection Center. The *Escherichia coli* W25K strain was isolated from a piglet with diarrhea as described previously [35]. The strains were cultured in LB medium to logarithmic growth period (OD = 0.5) and then were transferred to Mueller-Hinton Broth (MHB) medium for the minimum inhibitory concentration (MIC) or minimum bactericidal concentration (MBC) test.

### 4.2. Peptide Synthesis

The 7 AMPs were synthesized and purified by a Chinese peptide company (DgPeptides Co., Ltd., Hangzhou, China), and the sequences were confirmed via matrix-assisted laser desorption/ionization time-of-flight mass spectrometry (MALDI-TOF MS). The purity of the AMPs was higher than 95%, which was measured by reversed-phase high-performance liquid chromatography.

### 4.3. Cell Culture

IPEC-J2 cells were cultured at 37 °C in 100% humidity and 5% CO_2_ conditions with DMEM/F12 (Thermo, Waltham, MA, USA) supplemented with 5% fetal bovine serum (Gibco, Waltham, MA, USA) and 1% penicillin/streptomycin. The medium was changed every other day.

### 4.4. MIC/MBC Test

The MIC test was assessed according to the process of the Clinical and Laboratory Standards Institute. The bacterial strains were diluted with MHB medium, and the concentration was 5 × 10^5^ CFU/mL. Then, 180 μL bacterial suspension was transferred to 96-well cell plates. The AMPs were diluted with phosphate-buffered solution (PBS), then 20 μL AMPs was added to the bacterial suspension, and the final concentrations were 200, 100, 50, 25, 12.5, 6.25, 3.125, and 1.56 μg/mL. Then, the bacteria were cultured at 37 °C for 8 h.

### 4.5. Cell Vitality Assay

Thiazolyl blue tetrazolium bromide (MTT) was purchased from Sigma-Aldrich (St. Louis, MI, USA). The MTT was dissolved in PBS (5 mg/mL). The cytotoxicity of AMPs to IPEC-J2 cells was tested by using an MTT assay. A density of 1 × 10^5^ cells in 180 μL medium per well was seeded in 96-well plates (Corning, New York, NY, USA), and then 20 μL AMPs was added. The final concentrations were 200, 100, 50, 25, 12.5, 6.25, 3.125, were 1.56 μg/mL. After 8 h culture, 20 μL MTT was added, and then the cells were continued to culture for 4 h. The supernatant was discarded, and 150 μL DMSO was added. After 30 min shaking at room temperature, the absorbance was measured at a wavelength of 490 nm. The cell viability was calculated by using the following equation:

Cell viability = (OD_Control_ − OD_AMP_)/OD_Control_ × 100%

### 4.6. Quantifying Adhe7rent Bacteria

To calculate the number of adherent *E. coli*, *E. coli* were cocultured with IPEC-J2 cells in DMEM/high-glucose medium (no penicillin/streptomycin, no FBS). Then, the cells were washed with PBS six times and lysed with 1% Triton X-100 for 20 min at room temperature. Next, 5 μL lysates were plated on MacConkey agar plates overnight. The total number of bacteria was quantified as CFUs.

### 4.7. TER and Permeability Measurement

To evaluate the barrier integrity of IPEC-J2 cells, the transepithelial resistance (TER) and was measured, and a permeability assay was performed. The initial TER was tested before the cells were seeded. IPEC-J2 cells were then seeded in a Transwell membrane insert (12 mm diameter, 0.4 μm pore size, Corning) at a density of 7 × 10^5^ cells/well. Then, 200 μL and 500 μL medium was added to the apical and basal compartments, respectively. Cecropin A (12.5 μg/mL) was added to the apical and basal compartments. The TER values were measured every day by using an ohm-meter fitted with chopstick electrodes (Millipore ESR-2; Burlington, MA, USA). Before each test, the plates were placed at room temperature for 30 min. The TER was calculated by using the following equation:

TER (Ω·cm^2^) = (TER − TER_initial_) × 0.3

To evaluate the permeability of the monolayer intestinal cells, FITC-dextran was used. FITC-dextran (Sigma-Aldrich, St. Louis, MO, USA) was dissolved in PBS at 5 mg/mL. After the medium was discarded, 200 μL FITC-dextran was added to the apical compartment, and 500 μL PBS was added to the basal compartment and cultured for 2 h. Then, 100 μL liquid from each well was transferred to 96-well plates, and the absorbance was read at 480 nm excitation and 520 nm emission wavelengths. Then, the content of the FITC-dextran in the basal compartment was calculated using a standard curve.

### 4.8. qPCR

Total RNA was extracted using TRIzol reagent according to the manufacturer’s instructions. Concentration and purity of RNA was checked by using a NanoDrop 2000. cDNA was generated from 1 μg total RNA using a First Strand cDNA Synthesis Kit (Thermo Scientific, Waltham, MA, USA). Quantitative PCR (qPCR) was performed to quantify mRNA expression levels of IL-6, IL-8 and TNF-α relative to that of a housekeeping gene, glyceraldehyde-3-phosphate dehydrogenase (GAPDH) by using SYBR Green mix (ABI) according to the manufacturer’s instructions. The forward and reverse primers are shown in Table S3.

### 4.9. Western Blotting

The total protein was extracted with lysis buffer. The concentration of protein was tested by using a BCA protein assay kit (Thermo Scientific, Waltham, MA, USA) and mixed with 5× loading buffer. 20 μg protein sample was loaded in each well. The supernatant was then separated by 10% SDS-PAGE and transferred onto a PVDF membrane (Millipore, Burlington, MA, USA). After blocking with 5% skimmed milk powder, the membrane was incubated with the appropriate primary antibodies overnight at 4 °C, followed by incubation with a horseradish peroxidase (HRP)-conjugated secondary antibody for 2 h. Bands were detected using an ECL Western Blotting Substrate (Thermo Scientific, Waltham, MA, USA). Band intensity was quantified using ImageJ software. The primary antibodies including β-actin, p-MEk, MEK, p-ERK, ERK, CDX2 were purchased from Cell Signaling Technology (CST, Danvers, MA, USA). To specifically inhibit the phosphorylation of ERK, PD184352 (CST) was dissolved in DMSO and then used at a concentration of 10 μM for 48 h.

### 4.10. Cell Immunofluorescence Aassay

To test the expression and location of tight junction proteins (ZO-1, occludin and claudin-1) [31] and the cytoskeleton (F-actin), cell immunofluorescence was used. The IPEC-J2 cells were cultured and became confluent on the slide. After treatment, the cells were washed with PBS three times, fixed with 4% polyoxymethylene for 30 min, and then washed by PBS again three times. For F-actin staining, 0.5% Triton X-100 was added for 20 min. After that, 0.5% bovine serum albumin was added for 1 h, and then the primary antibodies were added and incubated with the cells at 4 °C overnight. The slides were then washed with PBST three times, and the FITC-labeled secondary antibody was added for 2 h. At last, the cell nuclei were stained by using DAPI (Santa Cruz Biotechnology, Dallas, TX, USA). The slides were then observed by using a fluorescence microscope. Primary antibodies including occludin (Abcam, Cambridge, UK), claudin-1 (CST, Danvers, MA, USA) and ZO-1 (Thermo Fisher Scientific, Waltham, MA, USA) were used. For staining F-actin, FITC-phalloidin (Sigma-Aldrich, St. Louis, MI, USA) was used.

### 4.11. Statistics

Data are expressed as the mean ± SEM. The Student’s *t*-test was conducted to determine the differences between 2 groups using SAS (version 9.2, SAS Institute Inc., Cary, NC, USA), and a one-way ANOVA was used to determine differences among groups. Differences were considered statistically significant when *p* < 0.05.

## Supplementary Materials

Supplementary materials can be found at http://www.mdpi.com/1422-0067/19/7/1941/s1.

## Abbreviations

IBD	Inflammatory bowel disease
AMP	Antimicrobial peptides
TJ	Tight junction
*E. coli*	*Escherichia coli*
IPEC-J2	Porcine jejunum epithelial cells
TER	Trans-epithelial electrical resistance
MEK	Mitogen-activated protein kinase kinase
ERK	Extracellular signal-regulated kinase
AA	Amino acid
JAMs	Junctional adhesion molecules
ZO	Zonula occludens
MAPK	Mitogen-activated protein kinase
