# Supplementary material for: Cecropin A Modulates Tight Junction-Related Protein Expression and Enhances the Barrier Function of Porcine Intestinal Epithelial Cells by Suppressing the MEK/ERK Pathway
Source: Int J Mol Sci. 2018 Jul 2;19(7):1941. doi: 10.3390/ijms19071941 (PMC6073479; doi:10.3390/ijms19071941)
Supplement: Supplementary file 1 [file ijms-19-01941-s001.pdf]

Supplementary Materials:

Cecropin A Modulates Tight Junction-Related Protein Expression and Enhances the Barrier Function of Porcine Intestinal Epithelial Cells by Suppressing the MEK/ERK Pathway

Table S1. The antibacterial ability of AMPs (MIC/MBC, μg/mL).

	BLP1	Cecropin A	PMAP-23	LL37	PG-1	PG-2	RMAD-6
E coli ATCC 35150（O157：H7）	100/100	6.25/6.25	100/100	50/50	3.13/12.5	6.25/6.25	ND
E coli SSI 82000	100/100	1.5/1.5	12.5/25	12.5/12.5	3.13/6.25	6.25/12.5	ND
E coli ATCC 25922	50/50	1.5/1.5	6.25/6.25	6.25/6.25	6.25/6.25	6.25/6.25	25/50
E·coli ATCC 35401 (O78:11）	50/50	3.125/6.25	25/25	12.5/25	6.25/6.25	12.5/12.5	50/50
E coli W25K	25/25	1.5/3.125	6.25/6.25	12.5/25	6.25/12.5	6.25/12.5	25/50
Salmonella paratyphoid B CMCC 50094	100/100	3.125/6.25	ND	50/50	3.125/6.25	12.5/50	ND
Salmonella enteritidis ATCC 9120	100/ND	1.5/1.5	ND	50/50	3.125/6.25	12.5/25	ND
Pseudomonas aeruginosa ATCC 27853	100/100	3.125/6.25	50/50	12.5/25	3.125/3.125	6.25/6.25	100/100

Pseudomonas aeruginosa ATCC 9027	100/100	3.125/3.125	25/25	12.5/25	3.125/3.125	6.25/12.5	50/50
Staphylococcus aureus 29213	25/25	50/100	3.125/6.25	50/50	1.5/3.125	6.25/6.25	6.25/12.5

MIC: Minimum inhibitory concentration; MBC: minimum bactericidal concentration. The results were confirmed by three independent experiments per treatment (n=3). Representative results of the three independent experiments are shown.

Table S2. The AMPs selected from different animal sources.

Name	Source	Length	Charge	3D structure	Sequence
Bombinin-like peptide 1 （BLP-1）	frog	28	3	α-helix	GIGASILSAGKSALKGLAKGLAEHFAN
Cecropin A	insect	37	7	α-helix	KWKLFKKIEKVGQNIRDGIIKAGPAVAVVGQATQIAK
PMAP-23	pig	24	6	α-helix	RIIDLLWRVRRPQKPKFVTVWVR
LL37	human	37	6	α-helix	LLGDFFRKSKEKIGKEFKRIVQRIKDFLRNLVPRTES
Protegrin-1	pig	18	7	disulfide bond	RGGRLCYCRRRFCVCVGR
Protegrin-2	pig	16	6	disulfide bond	RGGRLCYCRRRFCICV
RMAD-6	mammals	33	8	disulfide bond	RRTCRCRFGRCFRRESYSGSCNINGRISSLCCR

Length: the number of amino acids; charge: the positive charge of the AMPs; 3D structure: the 3D structure of the AMPs; sequence: the amino acid sequence of AMPs.

Table S3. The primer of IL-6, IL-8 and TNF-α for qPCR.

	Forward	Reverse	Tm（℃）
IL-6 pig	CAAAGCCACCACCCCTAAC	TCGTTCTGTGACTGCAGCTT	60
IL-8 pig	TTCTTCTTTATCCCCAAACTGG	CCACATGTCCTCAAGGTAGGA	60
TNF-α	CCAATGGCAGAGTGGGTATG	TGAAGAGGACCTGGGAGTAG	60

F: forward primer, R: reverse primer.

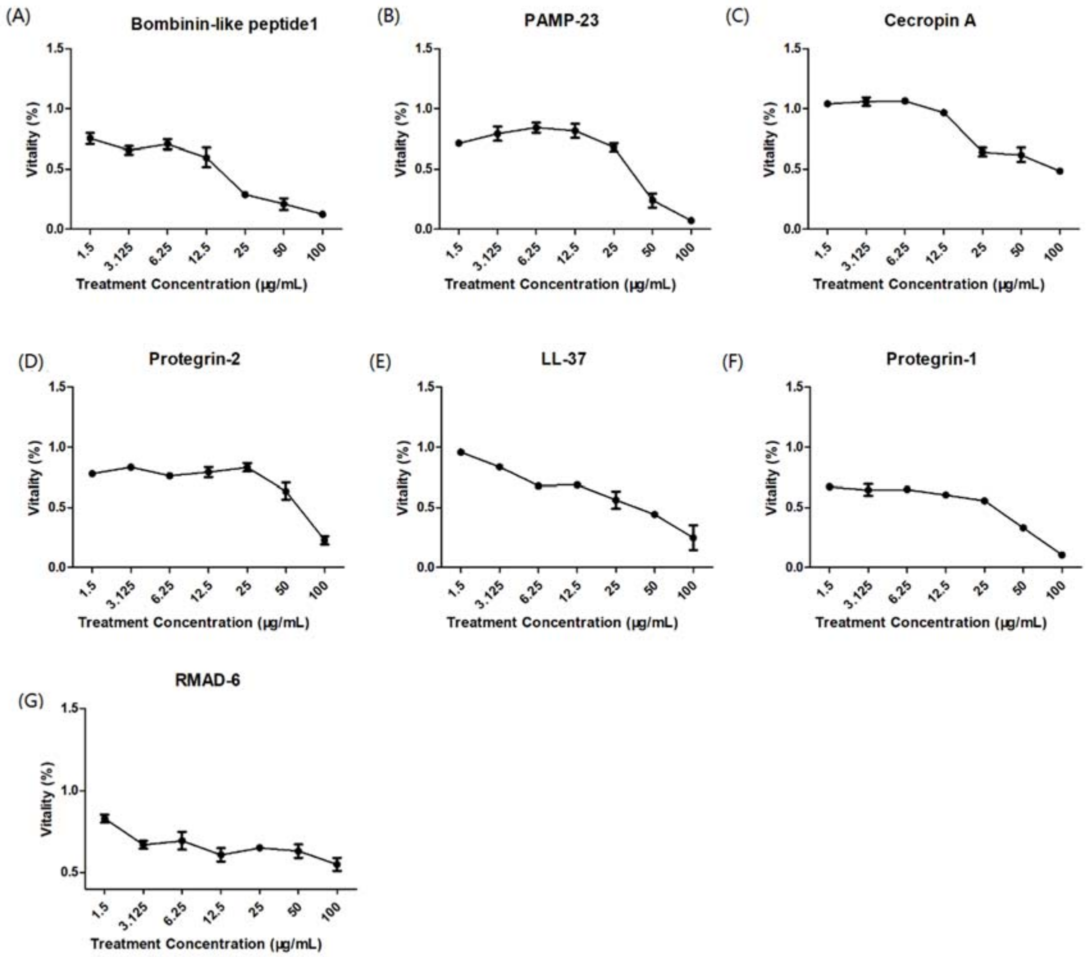

Figure S1. The effect of AMPs on IPEC-J2 cell vitality. The cell vitality was measured by using MTT assay after AMPs treated for 8h. The results were confirmed by three independent experiments per treatment (n = 6 for each experiment).
